# Thrombin related peptide TP508 promoted fracture repair in a mouse high energy fracture model

**DOI:** 10.1186/1749-799X-4-1

**Published:** 2009-01-29

**Authors:** Brain M Hanratty, James T Ryaby, Xiao-Hua Pan, Gang Li

**Affiliations:** 1 Department of Orthopaedic Surgery, School of Biomedical Sciences, Queen's University Belfast, 97 Lisburn Road, Belfast, BT9 7B, UK; 2 Research and Development, OrthoLogic Corp, 1275 West Washington Street, Tempe, AZ, USA; 3 Department of Orthopaedic Surgery, People's Hospital of Shenzhen City, Shenzhen, PR China; 4 Department of Orthopaedics & Traumatology, The Chinese University Hong Kong, Clinical Sciences Building, Prince of Wales Hospital, Shatin, Hong Kong, PR China

## Abstract

**Background:**

Thrombin related peptide (TP508) is a 23 amino-acid synthetic peptide that represents a portion of the receptor-binding domain of thrombin molecule. Previous studies have shown that TP508 can accelerate musculoskeletal tissue repair including fracture healing.

**Objectives:**

The aim of this study was to investigate the effect of TP508 on fracture healing in a murine fracture model representing high energy fracture situation.

**Methods:**

Eighty CD 1 mice underwent controlled quadriceps muscle crush and open transverse mid diaphyseal femoral fracture that was then fixed with an external fixator. Animals were randomised into four groups to receive an intra-operative dose of either 100 μg TP508 into the fracture gap; 100 μg TP508 into the surrounding damaged muscle tissues; 10 μg TP508 into the fracture gap, or control equal amount of saline into the fracture gap. Radiographic assessment was performed weekly for 5 weeks; histological analysis was at 3 and 5 weeks post fracture and biomechanical testing of the fractured bone was performed at 5 weeks post fracture.

**Results:**

Mechanical testing data showed that the fracture stiffness was significantly higher in the group receiving 100 μg TP508 into the fracture gap than other groups. Histological and radiographic analysis revealed a trend of increase in bone formation in the 100 μg TP508 injected into the fracture gap group compared to the saline control group. It was noted that the scar tissues was significantly less in Group II comparing with the saline control group and there was increased blood vessel formation in the crushed muscles and fracture gap areas in the groups receiving TP508 comparing to the saline control group.

**Conclusion:**

The results from this study demonstrated the use of thrombin related peptide TP508 in the situation of a high energy fracture can promote fracture healing and reduce the potential complications such as muscle fibrosis and fracture delayed or non-union.

## Background

Some 5–10% of patients that suffer a fracture throughout the world have problems with fracture healing. These include malunion, delayed union, non union, infection and avascular necrosis. After a fracture occurs the ability of a fracture to heal depends on several factors that include the systemic ability of the patient, the location of the fracture and the type of treatment received. Of the variables that can affect the rate of healing the amount of energy that causes the fracture has significance, the extent of injuries to the surrounding soft tissue may determine the fracture healing outcome. This is recognised by its inclusion in several scoring systems to help predict clinical outcomes and higher energy fractures are at greater risk of complications such as infection, delayed union or non-union.

Thrombin related peptide (TP508) represents one of the receptor binding domains of thrombin and several *in vitro *and *in vivo *studies have shown that TP508 had positive effects in the repair of the musculoskeletal tissues [1-3]. The positive effects of TP508 involve changes in the inflammatory response, enhancing cell recruitment and angiogenesis [4]. Since TP508 has been reported to promote fracture healing and the high energy fracture is always associated with soft tissue damages at the fracture sites, we hypothesized that administration of TP508 into the fracture site or into the damaged soft tissue site in a high-energy fracture model would benefit the fracture repair.

A mouse fracture model of delayed fracture healing similar to clinical conditions of high-energy fracture was originally described by Bunn et al [5] and was a development from a previous validated mouse open femur osteotomy models [6,7]. The aim of this study was to test the effectiveness of a single injection of TP508 given at time of surgery in the established mouse fracture model with controlled muscle crush that mimics high energy fracture healing.

## Methods

### Animal model of high energy fracture

**Figure 1:**
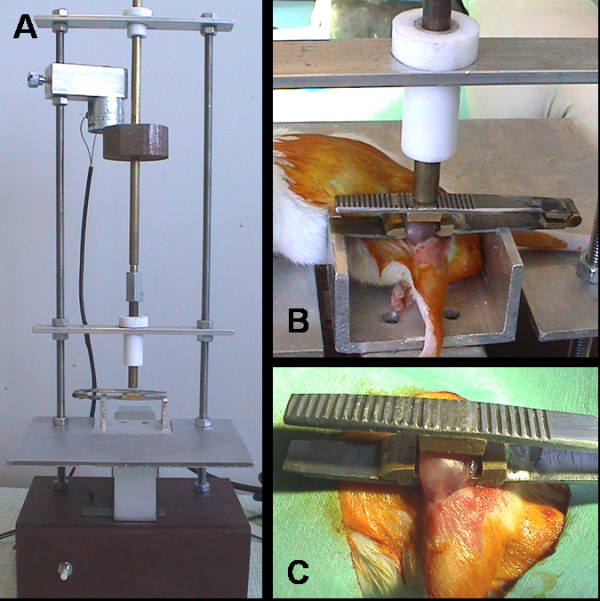
**A. Crushing jig**. **B**. Crush forceps in situ within crush jig. **C**. Custom made crush forceps.

3 month old CD1 mice were used with age ranging from 12–14 weeks and mean body weight of 39.75 +/- 3.026 g. General anaesthetisa was induced using 2% isoflurane in a nitrous oxide: oxygen (50:50) mixture at 400 ml/min in a sealed chamber. The skin was incised along the length of the femur from the left knee to the greater trochanter, the fascia lata was then incised and split distally starting from the prominent landmark of the adductor tubercle. The muscle bellies of the overlying quadriceps and hamstring muscles beneath were gently parted to gain access to the femoral diaphysis. The quadriceps muscle belly was crushed using a custom made crush forceps and crushing jig as previously described [5]. In brief, the crush forceps were passed either side of the quads muscle, and then the forceps and mouse were positioned in the muscle-crushing jig as shown in Fig. 1. A weight of 200 g was released from a 130 mm height to injure the quadriceps muscle and the crushed muscle was approximated to its original position on the femoral diaphysis. A femoral osteotomy was then performed according to the methods reported previously and fracture was fixed with an external fixtor as described before [6,7]. The skin was closed and a digital radiograph was carried out immediately to ensure correct fracture fixation.

Post operatively the animals were placed in individual cages and were recovered under heating lamps and mats in the first 24 hours. They were allowed unlimited cage activity until the day of termination.

### Randomisation and injection of TP508

To ensure no bias in the animal selection, a randomisation and coding was used to assign each animal to a group. When an individual animal was prepared for the operation it was given a numeric code from the list and allocated into whatever treatment group tagged to that code. This meant that the main investigator was blinded to the groups at time of outcome measurement.

There were 4 experimental groups and each contained twenty animals. At the time of surgery, Group I received an injection of 100 μg TP508 in 20 μl PBS into the fracture gap; Group II received an injection of 100 μg TP508 in 20 μl PBS into the surrounding damaged muscle; Group III received an injection of 10 μg TP508 in 20 μl PBS into the fracture gap and Group IV as the control group received an injection of 20 μl PBS saline into the fracture gap.

### Mechanical testing

After termination, the skin over both limbs was removed. The surrounding muscle then removed by sharp dissection, the quadriceps muscle carefully isolated, excised and preserved. Both femurs were disarticulated from the pelvis and knee joints taking care not to disrupt the structural integrity of the fracture site. The external fixators were removed by cutting through the pins using a diamond cutting disc and a haemostat to prevent pin spinning. The pin remnants were removed by gentle anti-clockwise rotation. Both femoral samples were then placed in a container with saline soaked gauze at room temperature 22°C and all mechanical tests performed within 4 hours post excision.

From each group 8 specimen pairs were tested to failure by 3-point bending using a 100 N load cell (Lloyd Instruments Ltd, UK). Each specimen was placed on two lower supports that were 9 mm apart and force applied at 5 mm/min at the mid-diaphysysis on the anterior surface such that the tension was in the posterior surface. Load displacement curves were generated and from these ultimate load and stiffness were determined for each specimen. The biomechanical properties of the fractured femur were expressed as a percentage of the contra lateral unfractured bone properties. In each instance the same person carried out the test. Every sample was coded so as to blind the investigator.

### Radiography analysis

Radiographs were taken at day 1, 7, 14, 21, 28 and 35 post-surgery. All the animals were anaesthetised and placed inside a high resolution digital radiography system (Faxitron MX-20, Faxitron X-ray Corporation, IL, USA). The facitron was calibrated before the procedure at a standard X-ray dose of 24 KV for 3 seconds at a distance of 12 cm. To control the plane of radiography a specifically made X-ray jig was attached to the external fixators via two portals in the crossbar. The animal was moved to the prone position on the jig, and placed centrally using the cross hairs for guidance. To monitor variations in x-ray beam penetration, an aluminium step-wedge phantom was attached to the jig and included in each radiograph taken. This technique meant that standardised lateral orthogonal x-rays were performed in an accurate and repeatable fashion.

Digital radiographs were taken in the TIFF format, coded and analysed by comparing the changes in pixel density across the fracture gap using UTHSCSA Image Tool program . Changes in pixel density corresponded with changes in bone mineralised tissue. Semi-quantitative analysis of the pixel density across the fracture gap was used and intra and inter observer variability measured using linearly weighted kappa and this showed a highly reproducible analysis. In brief five pixel density histograms were generated across the fracture gap and the pattern generated allocated a score of minus 1, 0 or plus 1 thus giving a maximum score for each radiograph of plus 5 and minimum of minus 5. Fracture callus size was measured and expressed as a ratio of the average femur diameter.

### Histology examination

**Figure 2:**
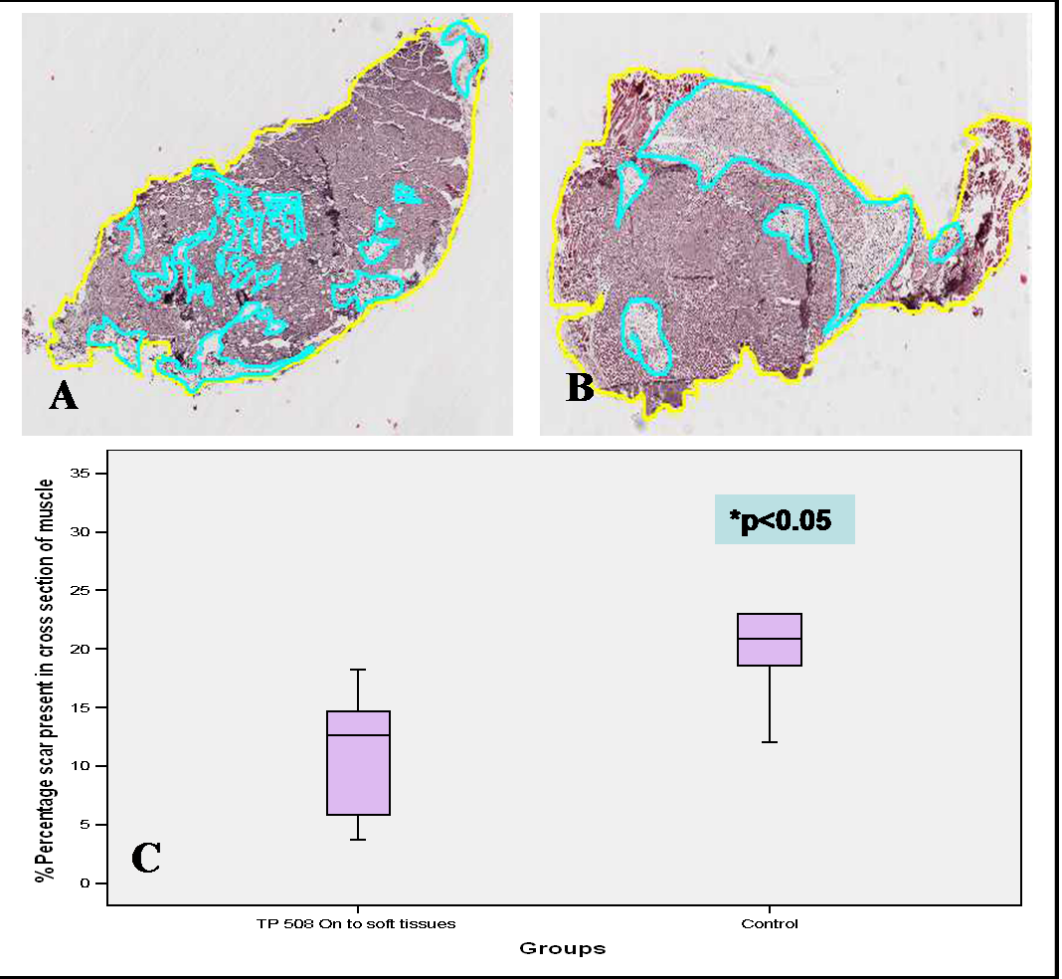
**A-C. Histological measurement of scar tissue present in cross sections of muscle taken from animals after three weeks**. Group II (**A**) had a mean of 13% and range 4–18% scar tissues, whereas group IV (**B**) had a mean of 22% and range of 14–23%. **C**. There was statistical difference between the groups (p = 0.016).

At 3 and 5 weeks post fracture, six animals from each group were sacrificed for histology examination. The femur was disarticulated from the pelvis and knee joints taking care to avoid disturbing the fracture callus. The bone specimen was coded and fixed for 48 h in 10% buffered formalin, then placed in 20% formic acid at 4°C for three weeks to decalcify. The specimen was ready when a green needle could pass easily through the bone. Decalcified samples were processed through graded alcohols, xylene and embedded in paraffin wax. The orientation was in the longitudinal plane such that all the fixator's holes were visible. 6 μm sections were cut, dewaxed in xylene and rehydrated through alcohol, then stained with haematoxtylin and eosin, and mounted using DPX. The quadriceps muscle specimens from each animal were also collected and embedded for histology analysis of scar tissue formation and blood vessels. Muscle from group II (100 μg injected into the soft tissues) was compared to that of group IV (Control). The area of scar tissue visualised on cross-sections of muscle was measured using image analysis software (Bioquant, Nova Version 4.00.8 Advanced Image Analysis, R&M Biometrics, Inc, USA) and expressed as a percentage of the total area of muscle cross-section (Fig 2). Blood vessels were immunostained by specific endothelial antigen marker CD31 on the paraffin sections as previously described [5] and the total number of blood vessels present in the fracture gap and crushed muscles was counted.

For digital photography, the slides were coded and a digital image of the fracture was taken using an Leica Microsystems camera and soft ware (Leica IM 50, Leica Microscopy Systems Ltd, Heerbrugg, Switzerland). The magnification was × 2.5 to ensure the whole of the fracture callus was included, and all pictures were taken the same sitting to ensure reproducibility. These images were transferred to Adobe Photoshop 7.0 (Adobe, San Jose, California, USA), and similar sized image showing only the fracture gap was cropped. Image analysis was carried out using image analysis software (Bioquant, Nova Version 4.00.8 Advanced Image Analysis, R&M Biometrics, Inc, USA). The amount of callus, fibrous tissue and cartilage in the fracture gap were quantified and compared.

### Statistical analysis

All quantitative data were transferred to the statistical program SPSS (Version 14, Chicago IL, USA). Analysis was carried out using non-parametrical tests, displaying distributions by means of boxplots and comparing groups with the Mann Whitney U test. Differences between groups were considered significant at p < 0.05.

## Results

### Aetiology

There were no statistically significant differences between the four groups of animals when comparing the age, weight and change in animal weight. During the experiment six animals died. Two animals, one from group I and another from group II did not survive anaesthesia when weekly radiographs were being taken, and another from group I had mechanical failure at week two and was killed humanly by terminal anaesthesia. Three animals from group IV did not regain nerve function in the operated limb following surgery and were killed humanly. These animals were replaced by littermates, so that each group contained twenty animals. Of the animals that survived the experiment, at the time of dissection 9 had non-union and two had evidence of mechanical failure of the external fixator. The animals that had non-union were made up of 4 from the control group, 2 from group II and 3 from group III. In the 2 animals of the non-unions from group III, there was evidence of deep infection.

### Radiographic assessments

**Figure 3:**
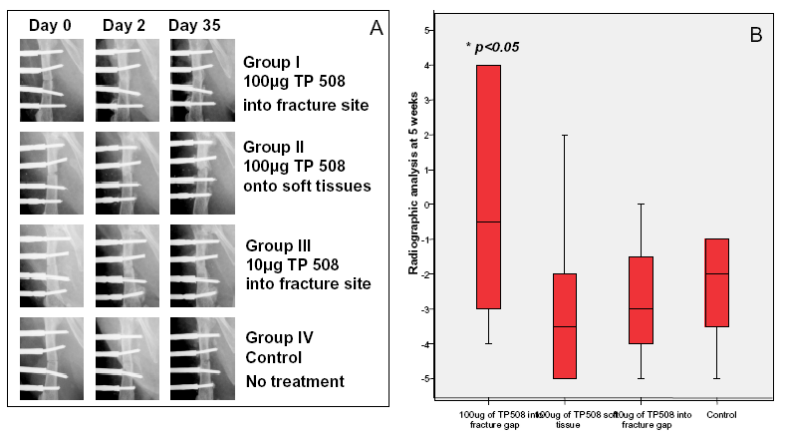
**A. Representative radiographs taken at day 0, 21 and 35 for the 4 experimental groups**. Group1 had shown a gradual improvement in callus formation in radiographic appearance through the time points. The fracture showed sign of union in Group I at day 35 post-fracture. There was no difference between Group II or III compared to Group IV at all the time points. **B**. Radiographic analysis data at week 5 post-fracture. Group I (100 μg TP508 injected in the fracture gap) had the largest amount of callus across the fracture gap compared to the other groups. Statistical analysis was carried out using non-parametrical Mann Whitney U test, difference between groups was considered significant at *p < 0.05.

There was no difference between the groups on day of surgery. Group I had shown the gradual improvement of bone formation in radiographic appearance through the time points (day 0 to day 35). The fracture showed sign of union in Group I at day 35 post-fracture. There was no difference between Group II or III compared to Group IV at all the time points (Fig 3A). The semi-quantitative analysis showed a delay in healing through out the five weeks in all groups. There was no difference between the groups in the first three weeks but at five weeks Group I differed in having more bone formation across the fracture gap, which was the only group to achieve a positive score (Fig 3B).

### Mechanical testing

**Figure 4:**
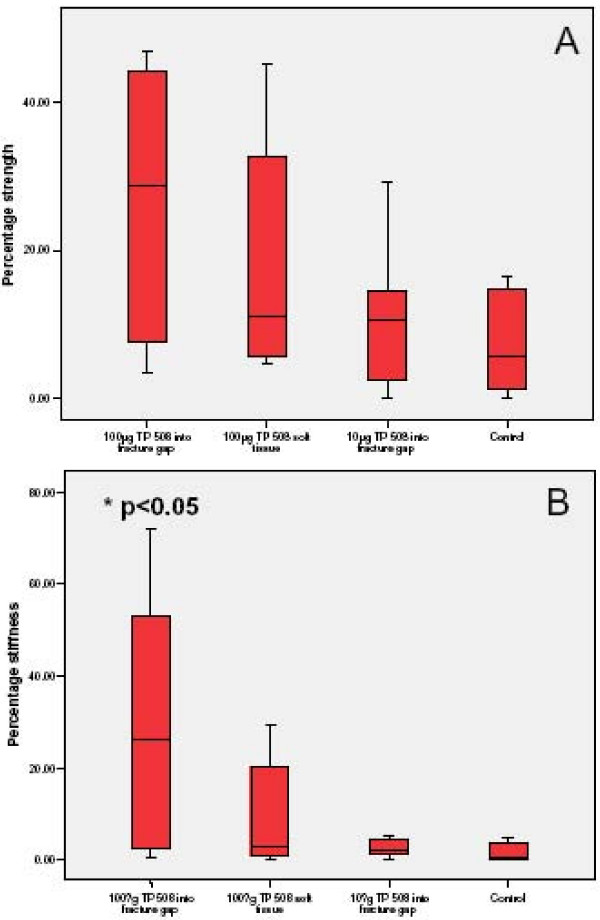
**A. Mechanical testing data of maximal load**. Properties were expressed as a percentage of maximal load to failure of the intact femur. This graph shows the strength ratio with Group 1 achieving up to 40% the strength of the contra-lateral intact bone, which is the highest among the groups.**B**. Mechanical testing data of stiffness. Properties were expressed as a percentage of the intact femur. This graph shows the stiffness ratio with Group 1 achieving up to 60% the stiffness of the contra-lateral bone, which was the highest among the groups and was significantly higher than that of the control group. Statistical analysis was carried out using non-parametrical tests, displaying distributions by means of boxplots and comparing groups with the Mann Whitney U test. Difference between groups was considered significant at *p < 0.05.

Results of the mechanical testing are shown in (Fig 4). The percentage ultimate loads showed no statistically significant difference between the groups but there was a trend of increasing strength towards Group I; the control group had less strength and stiffness compared to the other groups (Fig 4A). There were statistically significant differences in the percentage stiffness between Group I (100 μg TP508 injected into fracture site) and Group IV the control (p < 0.05); and there was no statistically significant differences between the other groups (Fig 4B).

### Histological analysis

**Figure 5:**
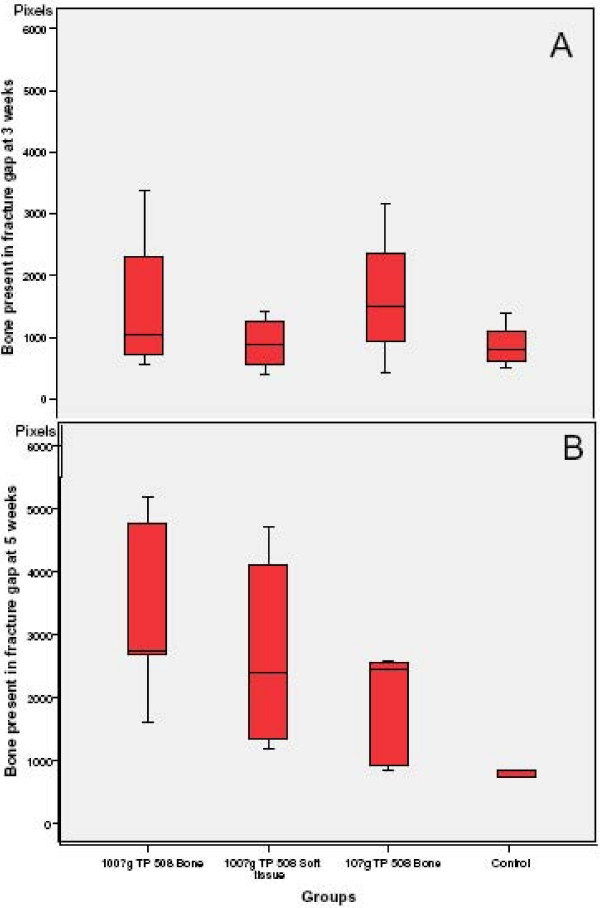
**A. At 3 weeks post-fracture, periosteal and endosteal callus in the fracture gap was greatest in Groups I and III**. Groups II and IV had mostly fibrous tissue and cartilage in the fracture gap at this time. **B**. At 5 weeks post-fracture, Group I and II had the most bone across the fracture gap followed by group III. Group IV had the least amount of bone in the fracture gap formed at this point.

On day 21 post-fracture, the amount of periosteal and endosteal callus (woven bone) in the fracture gap was greatest in Group I; periosteal callus was most evident in group I followed by Group III; Groups II and IV had mostly fibrous tissue and cartilage in the fracture gap at this time (Fig 5A). At day 35 post-fracture, Group I and II had the most bone across the fracture gap followed by group III; Group IV had the least amount of bone; periosteal callus was most evident in group II, and least in group IV (Fig 5B). The scar tissues were significantly reduced in Group II comparing with the control group (Fig 2A–C) and there was a trend of increased blood vessel formation in the crushed muscles and fracture gap areas in the groups receiving TP508 comparing to the saline control group (not shown).

## Discussion

In this study the synthetic peptide TP508 was tested in a mouse model mimicking high energy-fracture conditions with soft tissue injuries, and showed positive effects on enhancing fracture healing. The time to union in mouse fracture models is about 3 weeks [6,7], but in the current study, most of the animals in the control group did not achieve fracture union at 5 weeks, suggesting a delayed fracture union. However, the mechanical, radiographic and histological data demonstrated a superior fracture healing in the group receiving an injection of 100 μg TP508 into the fracture gap. This is in agreement with previous studies showing the benefit of TP508 in enhancing healing of various musculoskeletal tissues [8,9]. The group receiving 10 μg TP508 into the fracture gap did not lead to a significant improvement of the fracture healing, suggesting that the dose of TP508 administration is important. The positive effects of TP508 on tissue repair appear to be dose-dependent. Previous studies had used various doses of TP508, ranging from 0.1 μg in excision wounds in rats [10] to 300 μg in rabbit distraction osteogenesis studies [11]. In a rat closed femoral fracture study, Wang et al [8] noted a TP508 dose dependant increase in fracture strength, 1 μg TP508 group increased the fracture strength by 21% and 10 μg TP508 group by 36% relative to the control group. Since most of the studies have used TP508 in a soluble injection form and given at the same time as the injury, and only a small amount of TP508 could retain their bioactivities to the repair phases, therefore a higher dose of TP508 is needed to show the positive effects. Recently, studies have shown that TP508 given in a slow release microsphere form is more effective in enhancing bone repair and consolidation even at a reduced dose [12]. In the present study, we have used two doses of TP508 (100 and 10 μg/ml) in PBS delivery form based on the data from previous studies, and the data showed that the higher dose 100 μg/ml resulted in significant promoting effects of fracture healing. The use of controlled slow release form of TP508 with the same dose in the similar animal model will be the subject for future investigation.

We have also used one group where TP508 (100 μg/ml) was administrated into the crushed muscle and it was hoped that TP508 will help to reduce the adverse effects of the pro-inflammatory cytokines released from the traumatised muscles and enhance fracture healing. *In vitro *and *in vivo *studies have shown that TP508 altered the inflammatory response through an increase in the expression of IL-1 and IL-2 [4], and to recruit endothelial cells and osteoblasts through chemotaxis to the wounded areas [13-15]. Wang et al showed in a rat closed femoral fracture model, a single percutaneous injection of TP508 improved the resultant biomechanical properties of the healing fracture, and TP508 induced gene expression of early growth factors, inflammatory response modifiers and angiogenesis-related factors [8]. The immune genes and growth factors that have been down-regulated by TP508 included several MHC Class II genes, Interferon-γ, IL 1β receptor type 2, IL10 and IL12 [8]. The ability of TP508 to alter the immune response was also found in the dermal tissues, in several wound healing studies it was found that TP508 could suppress inflammatory responses at later stages of healing [1,10]. These findings are in agreement with those of Ryaby et al and Li et al who in a rat closed diaphyseal fracture mode [16] and in a rabbit distraction osteogenesis model [11,12] described a significant reduction in the number of inflammatory cells at the later stages of healing. Although there was no statistical difference between Group II and the control group in fracture callus volume and mechanical properties, there was significant reduction of scar tissue formation in the crushed muscles in group II, suggesting that TP508 may have a positive effect on muscle repair and regeneration, and this may in turn to facilitate soft tissue recovery and angiogenesis following high energy fracture. The use of TP508 to aid soft tissue healing needs future careful investigation.

As angiogenesis is an essential part of fracture repair [17] and early studies have noted that TP508 may have positive effect on angiogenesis. TP508 was shown to promote both the size and number of blood vessels in the chick chorioallantoic model [13] and TP508 enhances angiogenesis throug up-regulation of the c-Fos and c-Jun genes and not the VEGF or MMP-2 genes [14]. This agreed with Vartanian et al who used a model of angiogenic sprouting and showed that TP508 did not increase VEGF gene expression [18]. In their assay, TP508 stimulated angiogenic sprouting to an extent similar, to the intact thrombin molecule, but the proteolytically active receptor agonists had no effect on angiogenic sprouting, thus TP508 may promote angiogenesis through its non-proteolytic receptor pathways [18]. In the present study, we have found that there was increased blood vessel formation in the crushed muscles and fracture gap areas and significantly reduced scar formation in the groups receiving TP508 (regardless the dose) comparing to the saline control group, indicating that the enhancement of fracture repair by TP508 is partially associated with the enhanced angiogenesis induced by TP508.

In conclusion, local administration of TP508 (100 μg) into the fracture gap has promoted fracture repair in a mouse model of high-energy fracture. The effect appears to be dose dependent and is associated with reduced inflammation and enhanced angiogenesis in the surrounding soft tissues and in the fracture gap. TP508 may therefore be used to argument high energy fracture healing and more research work is needed to determine the best form and dose of TP508 delivery for its potential clinical applications.

## Competing interests

The authors declare that they have no competing interests.

## Authors' contributions

BMH carried out the animal experiments and participated in experimental design and the first draft of the manuscript. JTR helped with study design and discussion. XHP helped with animal experiments and study design. GL was involved in the study design and overall coordination, and was the grant holder. All authors read and approved the final manuscript.
